# 10-Hy­droxy-10-(1,3-thia­zol-2-ylmeth­yl)phenanthren-9(10*H*)-one

**DOI:** 10.1107/S1600536810022439

**Published:** 2010-06-16

**Authors:** Hoong-Kun Fun, Jia Hao Goh, Yang Liu, Yan Zhang

**Affiliations:** a X-ray Crystallography Unit, School of Physics, Universiti Sains Malaysia, 11800 USM, Penang, Malaysia; b School of Chemistry and Chemical Engineering, Nanjing University, Nanjing 210093, People’s Republic of China

## Abstract

In the title phenanthrenone compound, C_18_H_13_NO_2_S, the dihydro­phenanthrene ring system is not planar, with its central ring distorted to a screw-boat conformation. The essentially planar thia­zole ring [maximum deviation = 0.005 (1) Å] is inclined at an inter­planar angle of 23.36 (5)° with respect to the mean plane through the dihydro­phenanthrene ring system. In the crystal packing, inter­molecular O—H⋯N hydrogen bonds link the mol­ecules into infinite chains along the *a* axis. Weak inter­molecular C—H⋯π inter­actions further stabilize the crystal packing.

## Related literature

For general background to and applications of phenanthrenone derivatives, see: Bloom (1961 ▶); Kumagai *et al.* (1997 ▶); McClellan (1987 ▶); Meyer & Spengler (1905 ▶); Milko & Roithova (2009 ▶); Mustafa *et al.* (1956 ▶); Nel *et al.* (2001 ▶); Schuetzle *et al.* (1981 ▶); Shimada *et al.* (2004 ▶); Zhang *et al.* (2004 ▶). For ring conformations, see: Cremer & Pople (1975 ▶). For related structures, see: Jones *et al.* (2002 ▶); Li *et al.* (2003 ▶); Sun *et al.* (2007 ▶); Wang *et al.* (2003 ▶). For the stability of the temperature controller used for the data collection, see: Cosier & Glazer (1986 ▶).
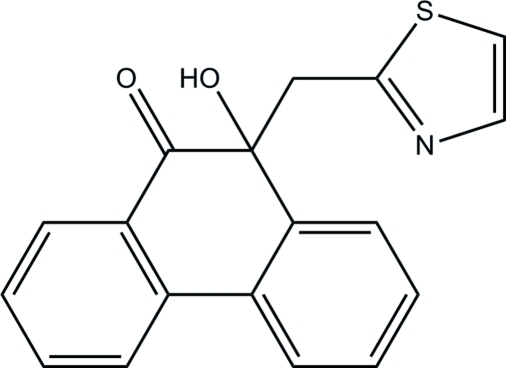


## Experimental

### 

#### Crystal data

C_18_H_13_NO_2_S
*M* *_r_* = 307.35
Orthorhombic, 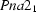

*a* = 12.5623 (17) Å
*b* = 7.3222 (10) Å
*c* = 15.462 (2) Å
*V* = 1422.3 (3) Å^3^
*Z* = 4
Mo *K*α radiation
μ = 0.23 mm^−1^
*T* = 100 K
0.33 × 0.17 × 0.17 mm

#### Data collection

Bruker APEXII DUO CCD area-detector diffractometer
Absorption correction: multi-scan (*SADABS*; Bruker, 2009 ▶) *T*_min_ = 0.926, *T*_max_ = 0.962
14577 measured reflections
3814 independent reflections
3635 reflections with *I* > 2σ(*I*)
*R*_int_ = 0.028

#### Refinement

*R*[*F* ^2^ > 2σ(*F* ^2^)] = 0.028
*wR*(*F* ^2^) = 0.074
*S* = 1.03
3814 reflections
203 parameters
1 restraint
H atoms treated by a mixture of independent and constrained refinement
Δρ_max_ = 0.32 e Å^−3^
Δρ_min_ = −0.21 e Å^−3^
Absolute structure: Flack (1983 ▶), 1674 Friedel pairs
Flack parameter: 0.04 (5)

### 

Data collection: *APEX2* (Bruker, 2009 ▶); cell refinement: *SAINT* (Bruker, 2009 ▶); data reduction: *SAINT*; program(s) used to solve structure: *SHELXTL* (Sheldrick, 2008 ▶); program(s) used to refine structure: *SHELXTL*; molecular graphics: *SHELXTL*; software used to prepare material for publication: *SHELXTL* and *PLATON* (Spek, 2009 ▶).

## Supplementary Material

Crystal structure: contains datablocks global, I. DOI: 10.1107/S1600536810022439/wn2393sup1.cif

Structure factors: contains datablocks I. DOI: 10.1107/S1600536810022439/wn2393Isup2.hkl

Additional supplementary materials:  crystallographic information; 3D view; checkCIF report

## Figures and Tables

**Table 1:** Hydrogen-bond geometry (Å, °) *Cg*1 and *Cg*2 are the centroids of C8–C13 and C2–C7 rings, respectively.

*D*—H⋯*A*	*D*—H	H⋯*A*	*D*⋯*A*	*D*—H⋯*A*
O2—H1*O*2⋯N1^i^	0.80 (2)	1.976 (19)	2.7542 (14)	165 (2)
C5—H5*A*⋯*Cg*1^ii^	0.93	2.83	3.6508 (16)	147
C12—H12*A*⋯*Cg*2^iii^	0.93	2.85	3.7214 (15)	156
C18—H18*A*⋯*Cg*1^iv^	0.93	2.72	3.3301 (16)	124

Symmetry codes: (i) 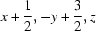; (ii) 
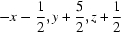
; (iii) 
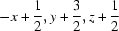
; (iv) 
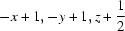
.

**Fig. 1.**
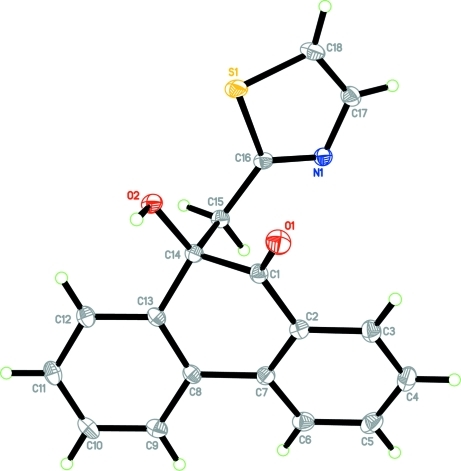
The molecular structure of the title compound, showing the atomic numbering scheme. Displacement ellipsoids are drawn at the 50% probability level. Hydrogen atoms are shown as spheres of arbitrary radius.

**Fig. 2.**
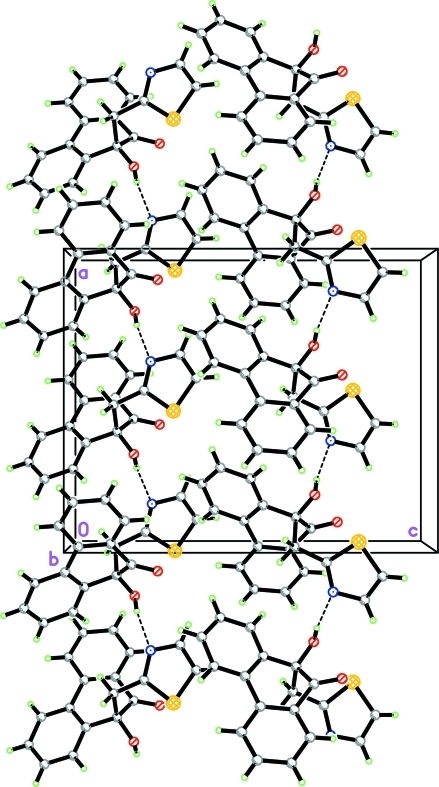
The crystal packing of the title compound, viewed along the b axis, showing hydrogen-bonded (dashed lines) one-dimensional chains along the a axis.

## supplementary crystallographic information

### Comment

Research interest in phenanthrenequinone can be traced back as early as 1905 (Bloom, 1961; Meyer & Spengler, 1905). Phenanthrenequinone and its derivatives possess a wide range of activities, especially biological and pharmaceutical. For example, phenanthrenequinone is one of the major quinones in diesel exhaust particles (Milko & Roithova, 2009), which plays a negative role in inducing pathogenic processes such as lung cancer (Schuetzle *et al.*, 1981), allergies (McClellan, 1987) or asthma (Nel *et al.*, 2001). Phenanthrenequinone has also been reported to be a good substrate for microsomal NADPH-cytochrome P450 reductase and that superoxide and hydroxyl radicals generated during redox cycling of the quinone by this flavin enzyme mainly participate in the DEP-prompted oxidative stress (Shimada *et al.*, 2004; Kumagai *et al.*, 1997). The photochemistry of phenanthrenequinone has been investigated early in 1956 (Mustafa *et al.*, 1956). In recent years, more complex products have been obtained in photoreactions of oxazoles with phenanthrenequinone (Zhang *et al.*, 2004). The crystal structures of 2-(4-hydroxy-3,5-dimethoxyphenyl)-1*H*-phenanthro[9,10-d]imidazole methanol solvate (Sun *et al.*, 2007) and 2,2,2-tris(cyclohexyloxy)-4,5-(2',2''-biphenylo)-1,3,2-dioxaphospholene (Jones *et al.*, 2002) have been reported. Due to the importance of phenanthraquinone derivatives, we report here the crystal structure of the title compound.

In the title compound (Fig. 1), the 1,2-dihydrobenzene ring (C1/C2/C7/C8/C13/C14) of the 9,10-dihydrophenanthrene ring system (C1-C14) is distorted towards a screw-boat conformation as observed in a previously reported structure (Wang *et al.*, 2003), with puckering parameters of Q = 0.4466 (13) Å, θ = 67.55 (18)° and φ = 320.40 (19)° (Cremer & Pople, 1975). In the 1,2-dihydrobenzene ring, atoms C1 and C14 deviate by 0.2034 (13) and -0.4508 (12) Å, respectively, in opposite directions from the mean plane through the remaining four atoms. The thiazole ring (C16/N1/C17/C18/S1) is essentially planar, with a maximum deviation of 0.005 (1) Å at atom C16. The interplanar angle formed between the thiazole ring and the mean plane through the 9,10-dihydrophenanthrene ring system is 23.36 (5)°. The geometric parameters are consistent with those observed in closely related 9,10-dihydrophenanthrenone structures (Wang *et al.*, 2003; Li *et al.*, 2003).

In the crystal packing, intermolecular O2—H1O2···N1 hydrogen bonds (Table 1) link the molecules into one-dimensional chains along the [100] direction (Fig. 2). The crystal packing is further stabilized by weak intermolecular C5—H5A···*Cg*1, C12—H12A···*Cg*2 and C18—H18A···*Cg*1 interactions (Table 1), where *Cg*1 and *Cg*2 are the centroids of C8-C13 and C2-C7 benzene rings, respectively.

### Experimental

The title compound was one of the products from the photoreaction between phenanthrenequinone (1 mmol) and 2-methylthiazole (4 mmol) in acetonitrile (50 ml). The compound was purified by flash column chromatography with ethyl acetate:petroleum ether (1:4) as eluents. X-ray quality single crystals of the title compound were obtained from slow evaporation of an acetone:petroleum ether (1:5) solution. *M.p.* 430–432 K.

### Refinement

Atom H1O2 was located in a difference Fourier map and allowed to refine freely [O2—H1O2 = 0.798 (19) Å]. The remaining H atoms were placed in calculated positions and were refined using a riding model, with C—H = 0.93 or 0.97 Å, *U*_iso_ = 1.2 *U*_eq_(C).

### Crystal data

**Table:**

C_18_H_13_NO_2_S	*F*(000) = 640
*M**_r_* = 307.35	*D*_x_ = 1.435 Mg m^−^^3^
Orthorhombic, *P**n**a*2_1_	Mo *K*α radiation, λ = 0.71073 Å
Hall symbol: P 2c -2n	Cell parameters from 7790 reflections
*a* = 12.5623 (17) Å	θ = 3.1–30.1°
*b* = 7.3222 (10) Å	µ = 0.23 mm^−^^1^
*c* = 15.462 (2) Å	*T* = 100 K
*V* = 1422.3 (3) Å^3^	Block, colourless
*Z* = 4	0.33 × 0.17 × 0.17 mm

### Data collection

**Table:**

Bruker APEXII DUO CCD area-detector diffractometer	3814 independent reflections
Radiation source: fine-focus sealed tube	3635 reflections with *I* > 2σ(*I*)
graphite	*R*_int_ = 0.028
φ and ω scans	θ_max_ = 30.1°, θ_min_ = 3.1°
Absorption correction: multi-scan (*SADABS*; Bruker, 2009)	*h* = −17→17
*T*_min_ = 0.926, *T*_max_ = 0.962	*k* = −10→9
14577 measured reflections	*l* = −17→21

### Refinement

**Table:**

Refinement on *F*^2^	Secondary atom site location: difference Fourier map
Least-squares matrix: full	Hydrogen site location: inferred from neighbouring sites
*R*[*F*^2^ > 2σ(*F*^2^)] = 0.028	H atoms treated by a mixture of independent and constrained refinement
*wR*(*F*^2^) = 0.074	*w* = 1/[σ^2^(*F*_o_^2^) + (0.0445*P*)^2^ + 0.2177*P*] where *P* = (*F*_o_^2^ + 2*F*_c_^2^)/3
*S* = 1.03	(Δ/σ)_max_ < 0.001
3814 reflections	Δρ_max_ = 0.32 e Å^−^^3^
203 parameters	Δρ_min_ = −0.21 e Å^−^^3^
1 restraint	Absolute structure: Flack (1983), 1674 Friedel pairs
Primary atom site location: structure-invariant direct methods	Flack parameter: 0.04 (5)

### Special details

**Table:**

Experimental. The crystal was placed in the cold stream of an Oxford Cryosystems Cobra open-flow nitrogen cryostat (Cosier & Glazer, 1986) operating at 100.0 (1)K.
Geometry. All esds (except the esd in the dihedral angle between two l.s. planes) are estimated using the full covariance matrix. The cell esds are taken into account individually in the estimation of esds in distances, angles and torsion angles; correlations between esds in cell parameters are only used when they are defined by crystal symmetry. An approximate (isotropic) treatment of cell esds is used for estimating esds involving l.s. planes.
Refinement. Refinement of F^2^ against ALL reflections. The weighted R-factor wR and goodness of fit S are based on F^2^, conventional R-factors R are based on F, with F set to zero for negative F^2^. The threshold expression of F^2^ > 2sigma(F^2^) is used only for calculating R-factors(gt) etc. and is not relevant to the choice of reflections for refinement. R-factors based on F^2^ are statistically about twice as large as those based on F, and R- factors based on ALL data will be even larger.

### Fractional atomic coordinates and isotropic or equivalent isotropic displacement parameters (Å^2^)

**Table:**

	*x*	*y*	*z*	*U*_iso_*/*U*_eq_	
S1	0.53518 (2)	0.49600 (5)	0.78827 (3)	0.02005 (8)	
O1	0.58478 (9)	0.98317 (13)	0.74430 (7)	0.0204 (2)	
O2	0.68596 (7)	0.68224 (13)	0.67503 (6)	0.01555 (18)	
N1	0.36431 (8)	0.62424 (14)	0.72334 (7)	0.0147 (2)	
C1	0.56431 (10)	0.94441 (17)	0.66980 (9)	0.0141 (2)	
C2	0.48837 (10)	1.05126 (17)	0.61503 (9)	0.0141 (2)	
C3	0.40687 (10)	1.14942 (18)	0.65511 (9)	0.0175 (2)	
H3A	0.4023	1.1531	0.7151	0.021*	
C4	0.33251 (11)	1.24171 (19)	0.60461 (10)	0.0204 (3)	
H4A	0.2769	1.3048	0.6307	0.025*	
C5	0.34165 (11)	1.23923 (19)	0.51504 (10)	0.0211 (3)	
H5A	0.2923	1.3021	0.4816	0.025*	
C6	0.42365 (10)	1.14409 (18)	0.47484 (9)	0.0181 (3)	
H6A	0.4289	1.1443	0.4148	0.022*	
C7	0.49871 (10)	1.04759 (17)	0.52450 (8)	0.0145 (2)	
C8	0.59038 (10)	0.95064 (18)	0.48531 (8)	0.0137 (2)	
C9	0.62580 (11)	0.98991 (18)	0.40142 (9)	0.0165 (2)	
H9A	0.5878	1.0717	0.3673	0.020*	
C10	0.71727 (10)	0.90792 (19)	0.36852 (9)	0.0182 (3)	
H10A	0.7404	0.9362	0.3130	0.022*	
C11	0.77403 (10)	0.78396 (19)	0.41853 (9)	0.0178 (2)	
H11A	0.8355	0.7303	0.3967	0.021*	
C12	0.73877 (10)	0.74002 (18)	0.50145 (9)	0.0155 (2)	
H12A	0.7761	0.6554	0.5345	0.019*	
C13	0.64729 (9)	0.82280 (17)	0.53514 (8)	0.0133 (2)	
C14	0.60853 (9)	0.77241 (17)	0.62538 (8)	0.0129 (2)	
C15	0.51421 (10)	0.63447 (17)	0.61865 (8)	0.0141 (2)	
H15A	0.4604	0.6844	0.5804	0.017*	
H15B	0.5398	0.5216	0.5933	0.017*	
C16	0.46450 (9)	0.59352 (16)	0.70480 (8)	0.0136 (2)	
C17	0.34056 (11)	0.56729 (19)	0.80632 (9)	0.0183 (3)	
H17A	0.2727	0.5786	0.8298	0.022*	
C18	0.42275 (12)	0.49388 (19)	0.85133 (10)	0.0196 (3)	
H18A	0.4188	0.4494	0.9076	0.024*	
H1O2	0.7346 (14)	0.751 (3)	0.6817 (12)	0.021 (4)*	

### Atomic displacement parameters (Å^2^)

**Table:**

	*U*^11^	*U*^22^	*U*^33^	*U*^12^	*U*^13^	*U*^23^
S1	0.01201 (13)	0.02777 (17)	0.02038 (16)	0.00009 (11)	−0.00259 (13)	0.01058 (13)
O1	0.0226 (5)	0.0242 (5)	0.0144 (5)	0.0006 (4)	−0.0024 (4)	−0.0021 (4)
O2	0.0109 (4)	0.0177 (4)	0.0181 (4)	−0.0014 (3)	−0.0036 (3)	0.0040 (3)
N1	0.0123 (5)	0.0154 (5)	0.0162 (5)	−0.0003 (4)	−0.0006 (4)	0.0018 (4)
C1	0.0112 (5)	0.0160 (5)	0.0151 (6)	−0.0008 (4)	0.0008 (4)	0.0009 (5)
C2	0.0136 (5)	0.0141 (6)	0.0146 (6)	−0.0009 (4)	−0.0010 (4)	−0.0002 (4)
C3	0.0171 (6)	0.0169 (6)	0.0186 (6)	−0.0006 (5)	0.0023 (5)	−0.0023 (5)
C4	0.0161 (6)	0.0169 (6)	0.0283 (7)	0.0022 (5)	0.0013 (5)	−0.0031 (5)
C5	0.0172 (6)	0.0177 (6)	0.0283 (7)	0.0014 (5)	−0.0039 (5)	0.0035 (6)
C6	0.0176 (6)	0.0191 (6)	0.0175 (6)	−0.0013 (5)	−0.0028 (5)	0.0031 (5)
C7	0.0130 (5)	0.0133 (5)	0.0172 (7)	−0.0012 (5)	0.0001 (5)	0.0009 (4)
C8	0.0121 (5)	0.0158 (6)	0.0132 (6)	−0.0023 (4)	0.0002 (4)	−0.0005 (4)
C9	0.0159 (6)	0.0192 (6)	0.0145 (6)	−0.0037 (4)	−0.0014 (5)	0.0018 (5)
C10	0.0160 (6)	0.0253 (7)	0.0135 (6)	−0.0069 (5)	0.0023 (5)	−0.0007 (5)
C11	0.0122 (5)	0.0221 (6)	0.0191 (6)	−0.0032 (4)	0.0018 (5)	−0.0042 (5)
C12	0.0124 (5)	0.0175 (6)	0.0167 (6)	−0.0023 (4)	−0.0003 (4)	−0.0019 (4)
C13	0.0118 (5)	0.0147 (5)	0.0134 (6)	−0.0036 (4)	0.0003 (4)	−0.0009 (4)
C14	0.0107 (5)	0.0152 (5)	0.0128 (5)	−0.0009 (4)	−0.0016 (4)	0.0009 (4)
C15	0.0113 (5)	0.0156 (6)	0.0155 (6)	−0.0019 (4)	−0.0009 (4)	0.0017 (4)
C16	0.0118 (5)	0.0144 (5)	0.0146 (6)	−0.0017 (4)	−0.0030 (4)	0.0027 (4)
C17	0.0157 (6)	0.0212 (6)	0.0181 (7)	−0.0007 (5)	0.0017 (5)	0.0022 (5)
C18	0.0170 (6)	0.0262 (7)	0.0156 (7)	−0.0048 (5)	−0.0008 (5)	0.0072 (5)

### Geometric parameters (Å, °)

**Table:**

S1—C18	1.7164 (16)	C7—C8	1.4823 (18)
S1—C16	1.7217 (13)	C8—C9	1.4011 (18)
O1—C1	1.2140 (17)	C8—C13	1.4075 (18)
O2—C14	1.4041 (14)	C9—C10	1.3927 (19)
O2—H1O2	0.798 (19)	C9—H9A	0.9300
N1—C16	1.3103 (16)	C10—C11	1.3894 (19)
N1—C17	1.3818 (17)	C10—H10A	0.9300
C1—C2	1.4965 (18)	C11—C12	1.3942 (19)
C1—C14	1.5383 (18)	C11—H11A	0.9300
C2—C3	1.3961 (18)	C12—C13	1.3998 (17)
C2—C7	1.4060 (18)	C12—H12A	0.9300
C3—C4	1.393 (2)	C13—C14	1.5232 (17)
C3—H3A	0.9300	C14—C15	1.5605 (17)
C4—C5	1.390 (2)	C15—C16	1.5014 (18)
C4—H4A	0.9300	C15—H15A	0.9700
C5—C6	1.390 (2)	C15—H15B	0.9700
C5—H5A	0.9300	C17—C18	1.3562 (19)
C6—C7	1.4064 (18)	C17—H17A	0.9300
C6—H6A	0.9300	C18—H18A	0.9300
			
C18—S1—C16	90.30 (7)	C9—C10—H10A	119.9
C14—O2—H1O2	107.7 (13)	C10—C11—C12	119.98 (12)
C16—N1—C17	111.03 (11)	C10—C11—H11A	120.0
O1—C1—C2	123.35 (12)	C12—C11—H11A	120.0
O1—C1—C14	122.59 (12)	C11—C12—C13	120.21 (12)
C2—C1—C14	113.93 (11)	C11—C12—H12A	119.9
C3—C2—C7	121.31 (12)	C13—C12—H12A	119.9
C3—C2—C1	119.04 (12)	C12—C13—C8	120.08 (12)
C7—C2—C1	119.64 (12)	C12—C13—C14	119.90 (11)
C4—C3—C2	119.52 (13)	C8—C13—C14	120.01 (11)
C4—C3—H3A	120.2	O2—C14—C13	113.16 (10)
C2—C3—H3A	120.2	O2—C14—C1	113.02 (10)
C5—C4—C3	119.81 (13)	C13—C14—C1	109.04 (10)
C5—C4—H4A	120.1	O2—C14—C15	104.96 (10)
C3—C4—H4A	120.1	C13—C14—C15	109.78 (10)
C4—C5—C6	120.89 (13)	C1—C14—C15	106.59 (10)
C4—C5—H5A	119.6	C16—C15—C14	112.70 (10)
C6—C5—H5A	119.6	C16—C15—H15A	109.1
C5—C6—C7	120.29 (13)	C14—C15—H15A	109.1
C5—C6—H6A	119.9	C16—C15—H15B	109.1
C7—C6—H6A	119.9	C14—C15—H15B	109.1
C2—C7—C6	118.17 (12)	H15A—C15—H15B	107.8
C2—C7—C8	119.20 (11)	N1—C16—C15	124.01 (11)
C6—C7—C8	122.56 (12)	N1—C16—S1	113.74 (10)
C9—C8—C13	118.81 (12)	C15—C16—S1	122.24 (9)
C9—C8—C7	121.79 (12)	C18—C17—N1	115.57 (12)
C13—C8—C7	119.30 (11)	C18—C17—H17A	122.2
C10—C9—C8	120.78 (13)	N1—C17—H17A	122.2
C10—C9—H9A	119.6	C17—C18—S1	109.36 (11)
C8—C9—H9A	119.6	C17—C18—H18A	125.3
C11—C10—C9	120.11 (12)	S1—C18—H18A	125.3
C11—C10—H10A	119.9		
			
O1—C1—C2—C3	−26.56 (19)	C7—C8—C13—C12	−175.24 (11)
C14—C1—C2—C3	149.37 (11)	C9—C8—C13—C14	−177.89 (11)
O1—C1—C2—C7	154.95 (13)	C7—C8—C13—C14	5.58 (17)
C14—C1—C2—C7	−29.12 (16)	C12—C13—C14—O2	16.31 (16)
C7—C2—C3—C4	1.73 (19)	C8—C13—C14—O2	−164.51 (11)
C1—C2—C3—C4	−176.73 (12)	C12—C13—C14—C1	143.01 (11)
C2—C3—C4—C5	−1.7 (2)	C8—C13—C14—C1	−37.82 (15)
C3—C4—C5—C6	0.7 (2)	C12—C13—C14—C15	−100.57 (13)
C4—C5—C6—C7	0.3 (2)	C8—C13—C14—C15	78.60 (14)
C3—C2—C7—C6	−0.76 (19)	O1—C1—C14—O2	−8.85 (17)
C1—C2—C7—C6	177.69 (11)	C2—C1—C14—O2	175.19 (10)
C3—C2—C7—C8	176.32 (11)	O1—C1—C14—C13	−135.62 (13)
C1—C2—C7—C8	−5.23 (18)	C2—C1—C14—C13	48.41 (13)
C5—C6—C7—C2	−0.27 (19)	O1—C1—C14—C15	105.94 (13)
C5—C6—C7—C8	−177.25 (12)	C2—C1—C14—C15	−70.03 (13)
C2—C7—C8—C9	−158.44 (12)	O2—C14—C15—C16	64.01 (13)
C6—C7—C8—C9	18.51 (19)	C13—C14—C15—C16	−174.07 (10)
C2—C7—C8—C13	17.98 (18)	C1—C14—C15—C16	−56.11 (13)
C6—C7—C8—C13	−165.07 (12)	C17—N1—C16—C15	177.95 (12)
C13—C8—C9—C10	−1.71 (19)	C17—N1—C16—S1	−0.74 (14)
C7—C8—C9—C10	174.73 (12)	C14—C15—C16—N1	120.47 (13)
C8—C9—C10—C11	0.73 (19)	C14—C15—C16—S1	−60.94 (14)
C9—C10—C11—C12	0.69 (19)	C18—S1—C16—N1	0.76 (11)
C10—C11—C12—C13	−1.11 (19)	C18—S1—C16—C15	−177.96 (11)
C11—C12—C13—C8	0.11 (19)	C16—N1—C17—C18	0.32 (17)
C11—C12—C13—C14	179.28 (11)	N1—C17—C18—S1	0.25 (16)
C9—C8—C13—C12	1.28 (18)	C16—S1—C18—C17	−0.54 (11)

### Hydrogen-bond geometry (Å, °)

**Table:**

Cg1 and Cg2 are the centroids of C8–C13 and C2–C7 rings, respectively.

**Table:**

*D*—H···*A*	*D*—H	H···*A*	*D*···*A*	*D*—H···*A*
O2—H1O2···N1^i^	0.80 (2)	1.976 (19)	2.7542 (14)	165 (2)
C5—H5A···Cg1^ii^	0.93	2.83	3.6508 (16)	147
C12—H12A···Cg2^iii^	0.93	2.85	3.7214 (15)	156
C18—H18A···Cg1^iv^	0.93	2.72	3.3301 (16)	124

Symmetry codes: (i) *x*+1/2, −*y*+3/2, *z*; (ii) −*x*−1/2, *y*+5/2, *z*+1/2; (iii) −*x*+1/2, *y*+3/2, *z*+1/2; (iv) −*x*+1, −*y*+1, *z*+1/2.
